# Endoscopic Retrograde Cholangiopancreatography Under General Anesthesia Compared to Conscious Sedation Study

**DOI:** 10.1093/jcag/gwad037

**Published:** 2023-10-03

**Authors:** Grant Greaves, Kaitlyn G Harding, Brent Parker, Vu C Nguyen, Azim Ahmed, Belinda Yee, Joël Perren, Mathew Norman, Morgan Grey, Rafael Perini, Fahd Jowhari, Adrian Bak

**Affiliations:** Orthopedic Surgery, University of Alberta Hospital, Edmonton, AB, Canada; General Surgery, Memorial University of Newfoundland, St John’s, NL, Canada; Provincial Health Services Authority, Kelowna, BC, Canada; Faculty of Medicine, University of British Columbia, Vancouver, BC, Canada; Faculty of Medicine, University of British Columbia, Vancouver, BC, Canada; Internal Medicine, University of Saskatoon, Saskatoon, SK, Canada; Pediatrics, Memorial University of Newfoundland, St John’s, NL, Canada; Faculty of Medicine, University of British Columbia, Vancouver, BC, Canada; Kelowna Gastroenterology Associates, Kelowna, BC, Canada; Kelowna Gastroenterology Associates, Kelowna, BC, Canada; Gastroenterology, Kelowna General Hospital, Kelowna, BC, Canada; University of British Columbia Okanagan, Southern Medical Program, Kelowna, BC, Canada; Kelowna Gastroenterology Associates, Kelowna, BC, Canada; Gastroenterology, Kelowna General Hospital, Kelowna, BC, Canada; University of British Columbia Okanagan, Southern Medical Program, Kelowna, BC, Canada; Kelowna Gastroenterology Associates, Kelowna, BC, Canada; Gastroenterology, Kelowna General Hospital, Kelowna, BC, Canada; University of British Columbia Okanagan, Southern Medical Program, Kelowna, BC, Canada

## Abstract

**Background:**

Endoscopic retrograde cholangiopancreatography (ERCP) is used to diagnose and treat pancreatic and biliary disease. The current standard is to conduct ERCP under conscious sedation (CS). Patient movement and agitation during ERCP under CS can result in procedure failure and complications. Aiming to reduce procedure failure rates and complications, Kelowna General Hospital (KGH) in British Columbia, Canada transitioned to performing ERCP under general anesthesia (GA) as the practice standard.

**Objective:**

To determine if conducting ERCP under GA compared to CS decreases procedure complications, particularly post-ERCP pancreatitis (PEP).

**Methods:**

The charts of 2,198 patients who underwent ERCP at KGH between 2015 and 2020 were reviewed. Before September 17, 2017, ERCP was performed under CS (*n* = 1,316). Afterwards, ERCP was conducted under GA (*n* = 882). Demographic, clinical, and procedural data were extracted. The data were analyzed using univariate and multivariate statistical analysis.

**Results:**

Procedure failure rates (CS = 9 percent, GA = 3 percent, *P* < 0.001) decreased in the GA cohort after adjusting for age, sex, and co-morbidities. Thirty-day mortality, intensive care unit (ICU) transfer, returns post-discharge, PEP, and cholangitis rates were similar between cohorts.

**Conclusion:**

Performing ERCP under GA compared to CS resulted in an increase in procedural success rates. Other complication rates were similar between groups.

## Introduction

Endoscopic retrograde cholangiopancreatography (ERCP) is a common procedure used to diagnose and treat conditions of the biliary tree and pancreas. The procedure involves inserting a long flexible camera (endoscope) through the mouth and into the first part of the small intestine to access the biliary system. Since its innovation in 1968,^1^ ERCP has evolved from a diagnostic procedure to one where complex therapeutic interventions are performed. A grading system for the complexity of common indications for ERCP highlights its evolution as it proposes four levels of procedural difficulty ranging from simple stone removal to complex pancreatic therapy.^2^ With its widespread use and increasing complexity, more research is needed on factors that affect procedural outcomes.

Compared to other endoscopic procedures, ERCP is a more lengthy and technically challenging procedure with a higher rate of complications.^3^ The most common complication is procedure failure. Procedural success depends on multiple factors including endoscopist experience, challenging anatomy,^4^ and patient agitation.^5^ Besides procedure failure, the most common complication of ERCP is post-ERCP pancreatitis (PEP).^6^ PEP can lead to pancreatic fluid collections, pseudocysts, necrosis, and life-threatening illness.^6,25^ Risk factors and mechanisms underlying PEP include both technical risk factors (difficult cannulation, repeated cannulation, pancreatic sphincterotomy, and pancreatic duct injection) and patient factors (history of PEP, recurrent pancreatitis, Sphincter of Oddi dysfunction, pre-operative pancreatic volume, and female sex).^26,27^ Protective factors include use of rectal non-steroidal anti-inflammatory drugs (NSAIDs).^28^ Other complications associated with ERCP include post-ERCP cholangitis (PEC), perforation, hemorrhage, cardiopulmonary complications, and mortality.^7^

Since ERCP is associated with significant morbidity and mortality, previous research has focused on identifying factors which may influence and particularly reduce complication rates. One potential explanation is that patients undergoing ERCP are generally sicker than patients undergoing other endoscopic procedures.^8^ Other bodies of research have concentrated on whether the choice of sedation (conscious sedation [CS] vs. general anesthesia [GA]) influences the rate of ERCP-related complications. CS is a drug-induced decrease in a patient’s level of consciousness through depression of the central nervous system providing anxiolysis and amnesia surrounding the procedure. GA is associated with an absolute loss of consciousness, a nulled response to painful stimuli, and complete muscle and reflex paralysis, including the loss of protective airway reflexes.^9^

Although there are no formal guidelines, in Canada ERCP is generally performed using CS unless the patient has had a previously failed ERCP.^10,11^ Due to the increasing complexity of ERCP, the potential for benefits to patient satisfaction (less intra-procedural movement and discomfort),^12^ improved technical success rate,^8^ and superior monitoring,^8,13^ there is an increased interest in the use of GA during ERCP procedures. Previous research has suggested that using GA is associated with lower rates of PEP and cardiopulmonary complications^14,15^ although this relationship has not been consistently documented.^16^

This retrospective chart review aims to expand the body of research focused on the relationship between ERCP-related complications and type of sedation (CS vs. GA). Because it is the most common serious complication of ERCP, PEP rates were chosen as the primary outcome of this study, with other outcomes including procedural failure also measured as secondary outcomes. Therefore, the aim of this study is to determine if ERCP performed under GA results in decreased PEP rates compared to CS, as decreased PEP rates suggest improved patient safety and would support a widespread change in anesthesia modality for ERCP.

## Materials and methods

### Study design

This study was a pre-post retrospective, single-institution review conducted at Kelowna General Hospital (KGH) in British Columbia, Canada, following a practice change in October 2017 where the standard of practice for sedation during performance of ERCP transitioned from CS with only occasional use of GA to the exclusive use of GA for all ERCP procedures. In 2020, a preliminary evaluation demonstrated that the performance of ERCP under GA was associated with fewer reported patient safety learning system events.^17^ This work was extended to this retrospective study to evaluate the effect of this practice change on procedural outcomes and recognized complications of ERCP.

### Ethics

Ethics approval was obtained from the University of British Columbia Clinical Research Ethics Board (REB) (H20-01296-A001) and Interior Health Research Ethics Board (REB) (2020-21-013-H).

### Inclusions and exclusion criteria

Individuals under the age of 19 years were excluded because this work primarily influences the adult population. The two months before and after the implementation of the practice change were excluded from the analysis because this was a period of adjustment for the program, which may have altered the analysis results. Only patients of two gastroenterologists (RP and AB) who managed patients over the entire course of the practice change were included to reduce confounders created by operator-specific variance in performance.

### Patient selection

All patients meeting the study inclusion criteria were identified through the support of a KGH analyst with the ability to query from the regional health authority’s surgical patient management system. This data was cross-referenced to the physicians’ office Electronic Medical Record platform (QHR Accuro) using a keyword search of terms like “ERCP” to aggregate all patients.

### Variables

Key outcomes collected include PEP, procedure failure, PEC, intensive care unit (ICU) transfer, 30-day return post-discharge, and mortality. These metrics were transcribed into a database by the study investigators.

### Data sources and measurement

The study investigators reviewed the medical charts from Accuro and the hospital’s electronic medical records system to extract the demographic, clinical, and procedural data. Medical charts were reviewed by five independent reviewers, and discrepancies or concerns were reviewed and reconciled by the senior investigator and senior gastroenterologist.

### Bias

In order to mitigate bias, gastroenterologists responsible for conducting the procedures were not involved in chart review except were discrepancies or questions of a technical nature were involved.

### Study size

This study was designed to be adequately powered to determine whether the use of GA for ERCP versus CS results in a statistically and clinically significant difference in PEP. Previous studies have reported PEP to have an incidence of 3 percent^18^ to 15 percent.^8^ It was agreed from these studies and a preliminary quality review of approximately 300 patients that PEP rates would be less than 20 percent in both cohorts. With this upper threshold in mind, we determined that an absolute PEP rate difference of 5 percent would be clinically relevant. Based on this assumption, for the study to be adequately powered, a minimum sample size of 903 in each arm was required for the study to be adequately powered using standard sample size estimates{Sealed, 2012, Power calculator for binary outcome superiority trial}.^19^ Given that approximately 1,000 ERCP procedures are performed each year at KGH and that some patients would be excluded from the study based on the below-described inclusion and exclusion criteria, we estimated that patients in the 2 years before and after the practice change would be reviewed to meet or exceed the minimum sample size.

### Statistical methods

Using univariate analysis, the non-adjusted and adjusted differences in rates of outcomes were calculated. Univariate analysis was conducted in Microsoft Excel program using Fischer’s exact tests and Chi-squared tests.

Multivariate logistic regression was also used to evaluate the effect of procedure change on outcomes (PEP, PEC, return post-discharge, ICU transfer, and 30-day mortality). The co-variates used were age, sex, and Charlson Comorbidity Index (CCI) score. The CCI is a validated method for estimating mortality risk due to comorbid disease.^20^ The Omnibus tests of model coefficients was used to determine whether the models are significant. The Nagelkerke and Cox and Snell *R* squared values are provided to explain how much of the variance is attributable to the model. The SPSS (version 29.0.0.0) program was used to conduct the multivariate analysis.

## Results

### Participants

Table 1 details patient demographic and clinical features. After reviewing inclusion and exclusion criteria, there were 1,316 patients in the CS cohort and 882 patients in the GA cohort. The mean patient age between cohorts was 65. Age distribution was similar between cohorts (*P* = 0.9), as was patient sex (females: *N*_CS_ = 719/1316 [55 percent], *N*_GA_ = 461/882 [53 percent], *P* = 0.3).

**Table 1.** Clinical and demographic patient information between cohorts.

Characteristics	Before practice change *N* (%)	After practice change *N* (%)	*P*-value
Total	1,316	882	
Age			0.939
19–20	4 (<1%)	1 (<1%)	
21–30	49 (4%)	35 (4%)	
31–40	68 (5%)	51 (6%)	
41–50	125 (9%)	68 (8%)	
51–60	214 (16%)	132 (15%)	
61–70	280 (21%)	224 (25%)	
71–80	316 (24%)	206 (23%)	
>81	260 (20%)	165 (19%)	
Sex			0.28
Female	719 (55%)	461 (52%)	
Male	597 (45%)	421 (48%)	
Location			0.90
Inpatient status	841 (64%)	566 (64%)	
Outpatient status	475 (36%)	316 (36%)	
Procedure indication			0.32
Bile duct stones	475 (36%)	324 (37%)	
Obstructive jaundice	164 (12%)	149 (17%)	
Pancreatitis of unknown cause	63 (5%)	41(5%)	
Stent removal or exchange	50 (4%)	27 (3%)	
Biliary stricture or leaking	47 (4%)	27 (3%)	
Biliary or pancreatic ductal disease treatment or tissue sampling	46 (3%)	24 (3%)	
Suspicion for pancreatic cancer	40 (3%)	20 (2%)	
Sphincter of Oddi dysfunction or stenosis	23 (2%)	6 (1%)	
Other	408 (31%)	264 (30%)	

The distribution of daycare versus inpatient status was similar between cohorts (*N*_CS_ = 841/1316 [64 percent], *N*_GA_ = 566/882 [64 percent], *P* = 0.9). Procedure indications between cohorts were similar with the most common procedure indication in the CS cohort being bile duct stones (*N*_CS_ = 475/1316 [36 percent], *N*_GA_ = 324/882 [37 percent]) followed by obstructive jaundice (*N*_CS_ = 162/1316 [12 percent], *N*_GA_ = 149/882 [17 percent]) and pancreatitis of unknown cause (*N*_CS_ = 63/1316 [5 percent], *N*_GA_ = 41/88 [5 percent]) (*P* = 0.3).

### Procedure outcomes

**Figure 1.**
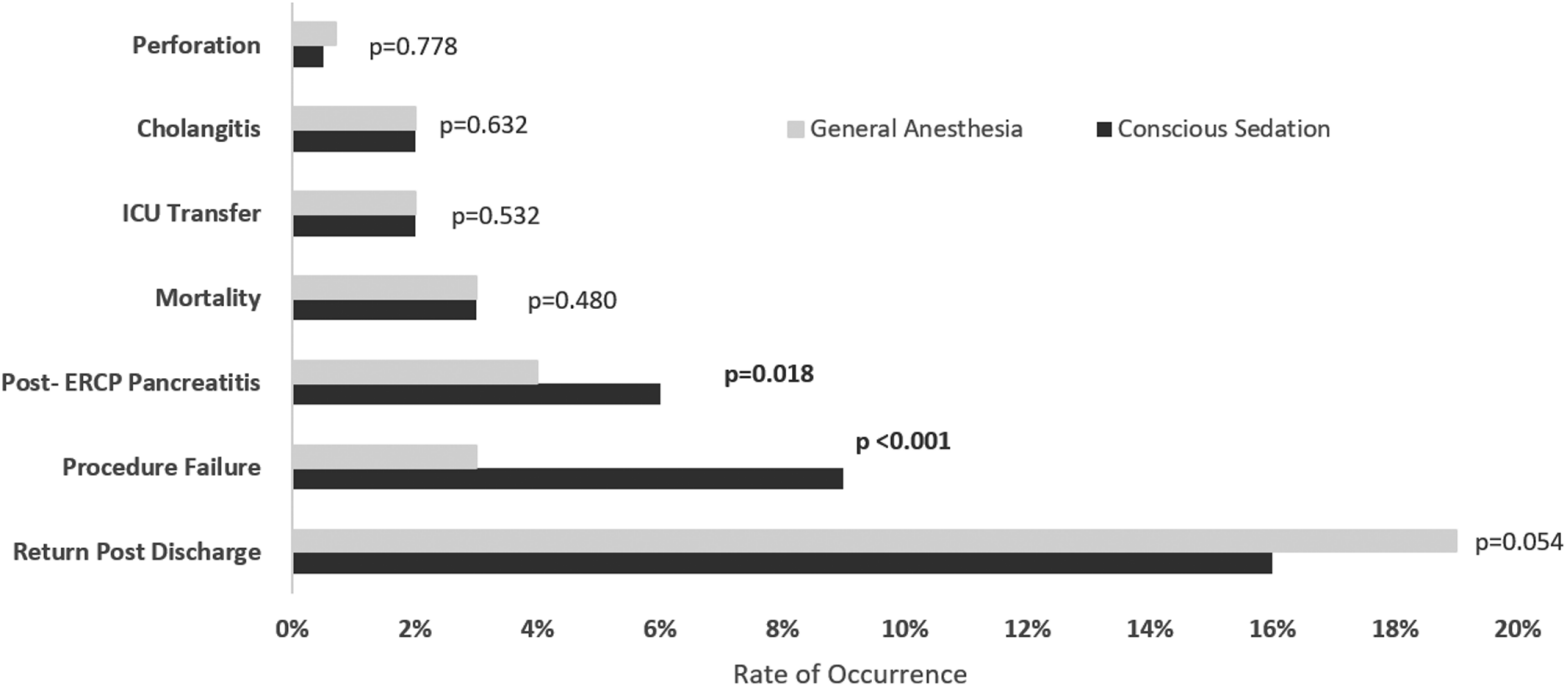
Difference in clinical outcomes between cohorts.

**Figure 2.**
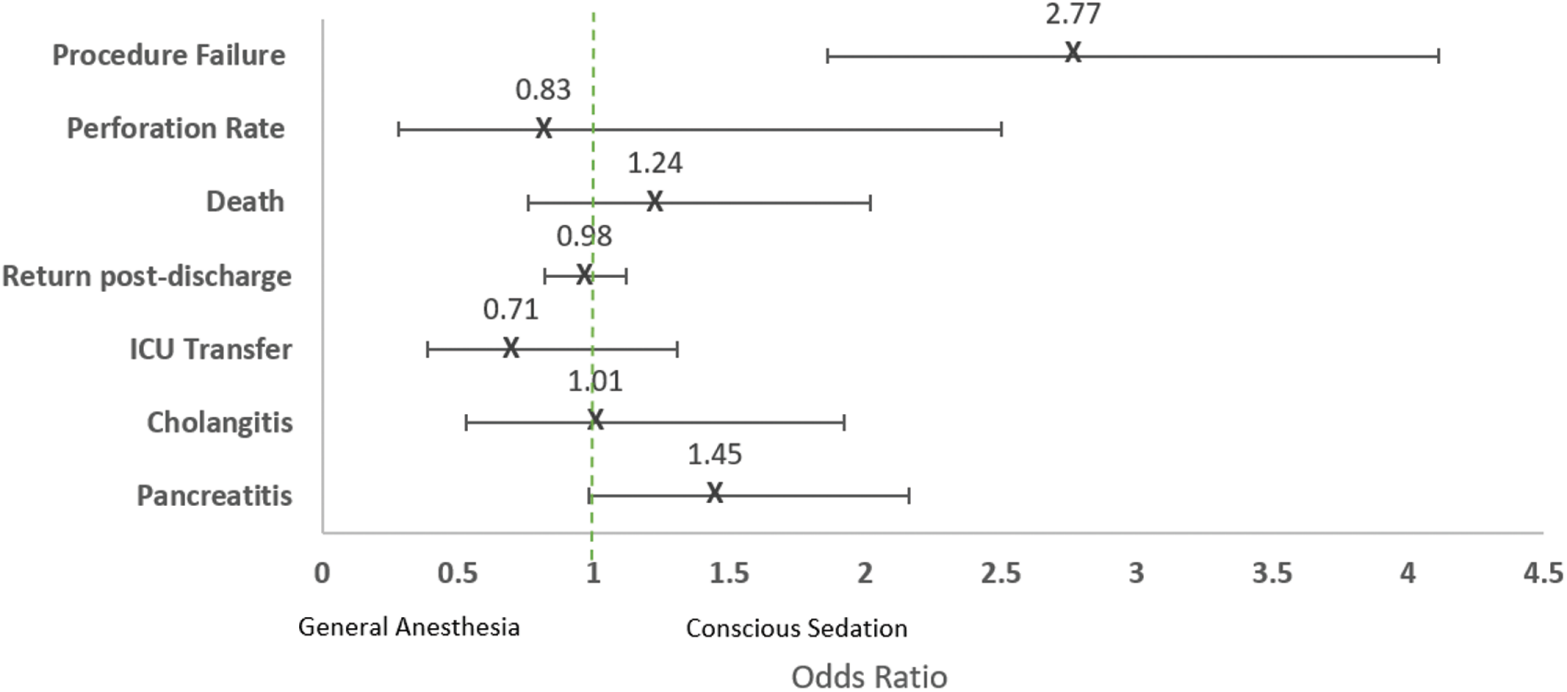
Odds ratios comparing difference between cohorts after adjusting for age, sex, and CCI score and 95 percent confidence intervals.

Univariate analysis outcomes are detailed in Figure 1. Figure 2 and Table 2 display multivariate analysis outcomes as measured in odds ratios (OR) comparing the outcomes in the two cohorts after adjusting for age, sex, and CCI score.

**Table 2.** Odds ratios for each dependent variable after procedure change are adjusted for age, sex, and Charlson Comorbidity Index score.

Variable	Odds ratio (OR)	95% CI	Expressed as probability (%)	*P*-value
Procedure failure^*^	2.77	1.86–4.11	73.48	<0.01
PEP	1.45	0.98–2.16	59.18	0.06
Cholangitis	1.01	0.53–1.92	50.25	0.99
ICU transfer	0.71	0.39–1.31	41.52	0.28
Return post-discharge	0.98	0.82–1.12	49.49	0.82
Death	1.24	0.76–2.02	55.36	0.40
Perforation rate	0.83	0.28–2.50	45.36	0.75

^*^Indicates statistically significant results.

Procedure failure rate was lower in the GA cohort (*N*_CS_ = 114/1316 [9 percent], *N*_GA_ = 24/882 [3 percent]), (OR 2.77, 95 percent CI 1.86–4.11, *P* < 0.01). The reasons for procedure failure varied significantly. In the CS cohort, 4 percent of patients experienced procedure failure due to agitation (*N* = 50/1,316) or abnormal structure (*N* = 8/1,316) while failure due to agitation did not occur in the GA cohort and failure due to abnormal structure was less than 1 percent (*N* = 2/882).

The PEP rates were similar between cohorts (OR 1.45, 95 percent CI 0.98–2.16, *P* = 0.06).

The rates of ICU transfer were similar, approximately 2 percent in both cohorts (*N*_CS_ = 28/1316, *N*_GA_ = 15/882, OR 0.71, 95 percent CI 0.39–1.31, *P* = 0.3).

The 30-day return post-discharge rates did not achieve statistical significance (*N*_CS_ = 205/1316 [16 percent], *N*_GA_ = 165/882 [19 percent], OR 0.98, 95 percent CI 0.82–1.12, *P* = 0.8). A larger sample size is required before a determination can be made.

The cholangitis rates were similar between cohorts (*N*_*CS*_ = 23/1,316 [2 percent], *N*_GA_ = 18/882 [2 percent], OR 1.01, 95 percent CI 0.53–1.92, *P* = 0.99).

The 30-day mortality rates were similar between cohorts (*N*_CS_ = 46/1316 [4 percent], *N*_GA_ = 26/882 [3 percent], OR 1.24, 95 percent CI 0.76–2.02, *P* = 0.40).

The perforation rates were similar between cohorts (*N*_CS_= 7/1316 [0.5 percent], *N*_GA_= 6/882 [0.7 percent], OR 0.83, 95 percent CI 0.28–2.50, *P* = 0.75).

## Discussion

The optimal anesthetic technique for ERCP has not been established. Previous research has suggested similar or lower ERCP-related complications when GA is used compared to CS. For instance, a 2005 chart review analyzed the outcomes of sixty-five patients who required GA and found no cardiopulmonary complications, and comparable sphincterotomy bleeding and PEP rates to CS.^15^ A 2014 retrospective chart review of 650 ERCPs performed in Michigan found significantly fewer cardiopulmonary complications and PEP rates.^14^ A 2002 retrospective chart review found procedure failure rates double in CS compared to GA.^11^ Our study adds to the evidence that ERCP is more successful under GA compared to CS, but that other complications may not be impacted by anesthesia modality.

Perforation, cholangitis, and 30-day mortality rates were also comparable with previous literature^21–23^ and between GA and CS groups in our study. As cholangitis is primarily due to inoculation of the biliary tree with bacteria from the gastrointestinal (GI) tract, its incidence is likely unrelated to anesthesia modality. Conversely, bowel perforation and PEP,^24^ in theory could be impacted by patient movement during ERCP under CS.^8^ Our data suggests that either anesthesia modality has little to no impact on these outcomes, or that the sample size was too small to elucidate statistically significant findings regarding these complications.

Consistent with previous research, the procedure failure rate was higher in the cohort who received CS compared to GA. This finding is understandable as patient movement and agitation, a common cause of procedure failure, is unlikely to occur under GA due to muscle and reflex paralysis.^9^ Insufficient airway protection has been reported in the literature as another cause of procedural failure in the patients undergoing ERCP with CS but was not observed in our study. Prevention of procedural failure is a benefit of GA in the performance of ERCP due to a reduced need for repeated procedures and uncomfortable interventions like dilations during the index procedure. PEP rates were similar between cohorts. However, our PEP rates were significantly lower than the pre-study estimates of up to 20 percent and the absolute difference between cohorts was lower than the 5 percent threshold required to be adequately powered. In other words, our study may have been inadequately powered to see a difference in PEP rates.

## Limitations

A comparison of types of GA was not conducted and may have demonstrated heterogeneity between methods. As a retrospective study, variability in the detail of procedural documentation resulted in imperfect data recording. The same two gastroenterologists performed all the pre- and post-practice change ERCPs included in the study, so we assumed that documentation practices were not significantly different in the two groups. The inclusion of patients of only two gastroenterologists in our study is both a strength (as it improved standardization and reliability of the data) and a weakness (as it decreases generalizability of the findings). Future multi-centre studies using a prospective cohort model with multiple endoscopists and a standardized procedural report template/form could standardize data reporting collection, thereby maintaining reliability of the data while improving generalisability. We did not assess if the patient position (supine, semi-prone, or prone) impacted our findings. Regardless, the data from this study clarify that GA results in fewer adverse outcomes than CS in the context of ERCP, which is in itself an important finding.

Our data did not include risk factors or protective factors for PEP. Given the retrospective nature of our study as well as funding limitations, it was not feasible to collect this data on all patients. Future studies should make note of them.

A cost analysis on the effect of transitioning to ERCP under GA fulltime was also not performed. Implementing the practice change at KGH required an increase in anesthesiology workload, as well as a development of a dedicated post-anesthetic recovery area (PAR) in the GI department staffed by PAR nurses. Any reduction in utilization or need for management of complications may offset the extra cost of provision of ERCP under GA, however, a formal cost analysis should be performed.

## Conclusion

Performing ERCP under GA compared to CS resulted in an increase in procedural success rates. Other complication rates were similar between groups.

## Supplementary Material

gwad037_suppl_Supplementary_Materials

## Data Availability

Deidentified individual participant data may be made available for a period of 5 years after the publication if the request is made. Requests may be made to mrbrentparker@gmail.com and are subject to the author’s data request policies and may be subject to an information sharing agreement and processing fees.
